# Relative effectiveness of influenza vaccines in elderly persons in the United States, 2012/2013-2017/2018 seasons

**DOI:** 10.1038/s41541-021-00373-w

**Published:** 2021-08-24

**Authors:** Marina Amaral de Avila Machado, Cristiano S. Moura, Michal Abrahamowicz, Brian J. Ward, Louise Pilote, Sasha Bernatsky

**Affiliations:** 1 Centre for Outcomes Research and Evaluation, Research Institute of the McGill University Health Centre, Montreal, QC H4A 3S5 Canada; 2 Department of Epidemiology, Biostatistics and Occupational Health, McGill University, Montreal, QC H3A 1A2 Canada; 3 Centre for Translational Biology, Research Institute of the McGill University Health Centre, Montreal, QC H4A 3J1 Canada

**Keywords:** Influenza virus, Outcomes research

## Abstract

Influenza immunization protects seniors against influenza and its potentially serious complications. It is uncertain whether standard-dose (SD) quadrivalent vaccine offers better protection over other formulations in the elderly. In this study, we compared the effectiveness of SD-trivalent, high-dose (HD) trivalent, SD-quadrivalent, and adjuvanted trivalent vaccines in seniors (≥65 years) in a real-world setting. We selected over 200,000 individuals in each of 6 influenza seasons from 2012 to 2018 using MarketScan® databases. The two outcomes were hospitalization or emergency room (ER) visit due to (1) influenza or (2) pneumonia. Here, SD-quadrivalent was associated with higher risk of influenza-related hospitalization/ER visit (adjusted hazard ratio (aHR) 1.14 and 95% confidence interval (95% CI) 1.05–1.24) and of pneumonia-related hospitalization/ER visit (aHR 1.04 and 95% CI 1.01–1.07) vs. HD-trivalent. SD-trivalent followed similar trends compared to HD-trivalent (aHR 1.16 and 95% CI 1.06–1.27 for hospitalized/ER visit influenza; aHR 1.07 and 95% CI 1.05–1.10 for hospitalized/ER visit pneumonia). We could not demonstrate risk differences between SD vaccine formulations and between adjuvanted trivalent and one of the other three vaccines. Risk estimates slightly varied across seasons. These findings suggest that SD vaccine formulations vs. HD-trivalent were associated with higher risk of hospitalization/ER visit for influenza and pneumonia in seniors.

## Introduction

Influenza-related complications leading to hospitalizations and death disproportionally affect seniors. The Centre for Disease Control (CDC) reported an estimated 38 million influenza cases during the 2019–2020 season, resulting in 400,000 related hospitalizations and 22,000 deaths^1^. Seniors account for 43% of influenza-related hospitalizations (a rate of 172.1 per 100,000) and 62% of deaths^1^.

Maintaining high influenza vaccination rates is crucial to protect the aging population^1–3^. Due to the novel coronavirus disease 2019 (COVID-19) virus and its continued surge, it is increasingly critical to provide influenza vaccination to this vulnerable population and minimize the burden on healthcare systems^4,5^. Unfortunately, studies have consistently shown that, compared to younger adults, seniors respond poorly to seasonal vaccination^3^. In addition to the standard-dose (SD) trivalent formulation (against two influenza type A and one type B strain), other vaccines have been developed to improve immunogenicity, including high-dose (HD) trivalent, adjuvanted trivalent, and SD-quadrivalent vaccines (against two different influenza type A and two type B strains)^3^. Previous studies have demonstrated trends of better protection with HD-trivalent than SD-trivalent, and the comparative effectiveness of preparations with adjuvanted trivalent, SD-quadrivalent, or HD-trivalent remains uncertain in real-world settings^6–11^. The CDC and many other national public health authorities have repeatedly emphasized that any available influenza vaccine is better than “no vaccine,” and timely vaccination is preferable to waiting for any specific vaccine^3,4^.

We urgently need real-world evidence of influenza vaccine outcomes in seniors to inform influenza vaccine program decisions in North America. In this study, we compared the effectiveness of SD-trivalent, HD-trivalent, SD-quadrivalent, and adjuvanted trivalent influenza vaccines in reducing the risk of hospitalization or emergency room (ER) visit for influenza and pneumonia in adults 65 years of age and older in the United States.

## Results

### Participant characteristics

The number of subjects ranged from 211,536 in 2017/2018 to 533,616 individuals in 2012/2013 (Table 1 and Supplementary Table 1). This does not reflect calendar differences in population vaccination rates but rather changes in the number of organizations supplying MarketScan with data. In the past few years, some employers have moved from the Medicare original insurance coverage to Medicare Advantage plans, which are not included in Marketscan. In our study, subjects were vaccinated with trivalent adjuvanted vaccine only in 2016/2017 and 2017/2018 seasons, and accounted for <5% of those immunized. In the 2012/2013 season, only HD-trivalent and SD-trivalent vaccines were available (Table 1). Over 60% of individuals had their influenza vaccine in September or October, and most seniors were female (55.0%). Diabetes (26.2%) and chronic obstructive pulmonary disease (16.2%) were the most frequent comorbidities. Details of baseline characteristics of each cohort are presented in Supplementary Tables 2–7.

**Table 1:** Number of subjects selected in each influenza season and distribution of influenza vaccine type.

Influenza season	Standard-dose trivalent	High-dose trivalent	Standard-dose quadrivalent	Adjuvanted trivalent	Total
2017/2018	23,965 (11.3%)	113,895 (53.8%)	64,615 (30.6%)	9061 (4.3%)	211,536 (100%)
2016/2017	99,217 (26.9%)	182,786 (49.6%)	81,808 (22.2%)	4480 (1.2%)	368,291 (100%)
2015/2016	142,075 (39.6%)	140,535 (39.1%)	76,578 (21.3%)	0	359,188 (100%)
2014/2015	229,071 (54.8%)	121,953 (29.2%)	66,861 (16.0%)	0	417,885 (100%)
2013/2014	369,326 (76.2%)	93,178 (19.2%)	21,837 (4.5%)	0	484,341 (100%)
2012/2013	457,740 (85.8%)	75,876 (14.2%)	0	0	533,616 (100%)

Among the events of influenza, 66.7% were hospitalizations and 33.3% were ER visits. Regarding pneumonia, 90.9% were hospitalizations and 9.1% were ER visits. Overall incidence rates of influenza-related hospitalization/ER visit varied across seasons; the rates were higher in 2017/2018 and lower in 2015/2016 and 2013/2014. Incidence rates for outcomes are presented in Supplementary Table 8.

### Pooled analyses

In the pooled analyses for each of the two outcomes, there were statistically significant differences between rates for subjects who received different vaccines, as indicated by the overall likelihood ratio test (LRT) (*p* < 0.004 for each outcome). Across all seasons and for both outcomes, SD-quadrivalent was associated with a higher risk than HD-trivalent, even if the corresponding adjusted hazard ratios (aHRs) were only marginally significant for hospitalized/ER visit pneumonia (aHR 1.14, 95% confidence interval (95% CI) 1.05–1.24 for hospitalized/ER visit influenza; aHR 1.04, 1.01–1.07 for hospitalized/ER visit pneumonia) (Table 2). Compared to HD-trivalent, SD-trivalent was associated with a higher risk of influenza-related hospitalization/ER visit (aHR 1.16, 1.06–1.27) and hospitalization/ER visit for pneumonia (aHR 1.07, 1.05–1.10) (Table 2). All comparisons of adjuvanted trivalent vs. other vaccines were not statistically significant (Table 2). In multivariable Cox models, the risk of influenza-related hospitalization/ER visit was higher for older subjects, men, residents of urban areas, those with hospitalization for pneumonia in the previous year, and baseline indicators of chronic obstructive pulmonary disease/asthma, congestive heart failure, and frail physical health, as well as previous use of bronchodilator inhalers and use of inhaled or oral corticosteroids (Supplementary Table 9).

**Table 2:** Adjusted hazard ratios and 95% confidence intervals for outcomes in pairwise comparisons (reference groups are indicated in the columns) in pooled analyses across all seasons (2012/2013–2017/2018).

Outcomes	High-dose trivalent	Standard-dose quadrivalent	Adjuvanted trivalent	Standard-dose trivalent^a^
Influenza (hospitalization/emergency room visit)				
High-dose trivalent	NA	0.87 (0.81–0.95)	0.98 (0.76–1.26)	0.86 (0.79–0.94)
Standard-dose quadrivalent	1.14 (1.05–1.24)	NA	1.12 (0.87–1.44)	0.98 (0.89–1.09)
Adjuvanted trivalent	1.02 (0.79–1.32)	0.90 (0.69–1.15)	NA	0.88 (0.67–1.15)
Standard-dose trivalent^a^	1.16 (1.06–1.27)	1.02 (0.92–1.12)	1.14 (0.87–1.48)	NA
Pneumonia (hospitalization/emergency room visit)				
High-dose trivalent	NA	0.96 (0.93–0.99)	0.98 (0.86–1.12)	0.93 (0.91–0.96)
Standard-dose quadrivalent	1.04 (1.01–1.07)	NA	1.02 (0.90–1.17)	0.97 (0.94–1.01)
Adjuvanted trivalent	1.02 (0.89–1.16)	0.98 (0.86–1.12)	NA	0.95 (0.83–1.09)
Standard-dose trivalent^a^	1.07 (1.05–1.10)	1.03 (0.99–1.06)	1.05 (0.92–1.21)	NA

^a^In pooled analyses, standard-dose trivalent vaccine was not considered in the 2016/2017 and 2017/2018 seasons.

### Season-specific analyses

**Fig. 1:**
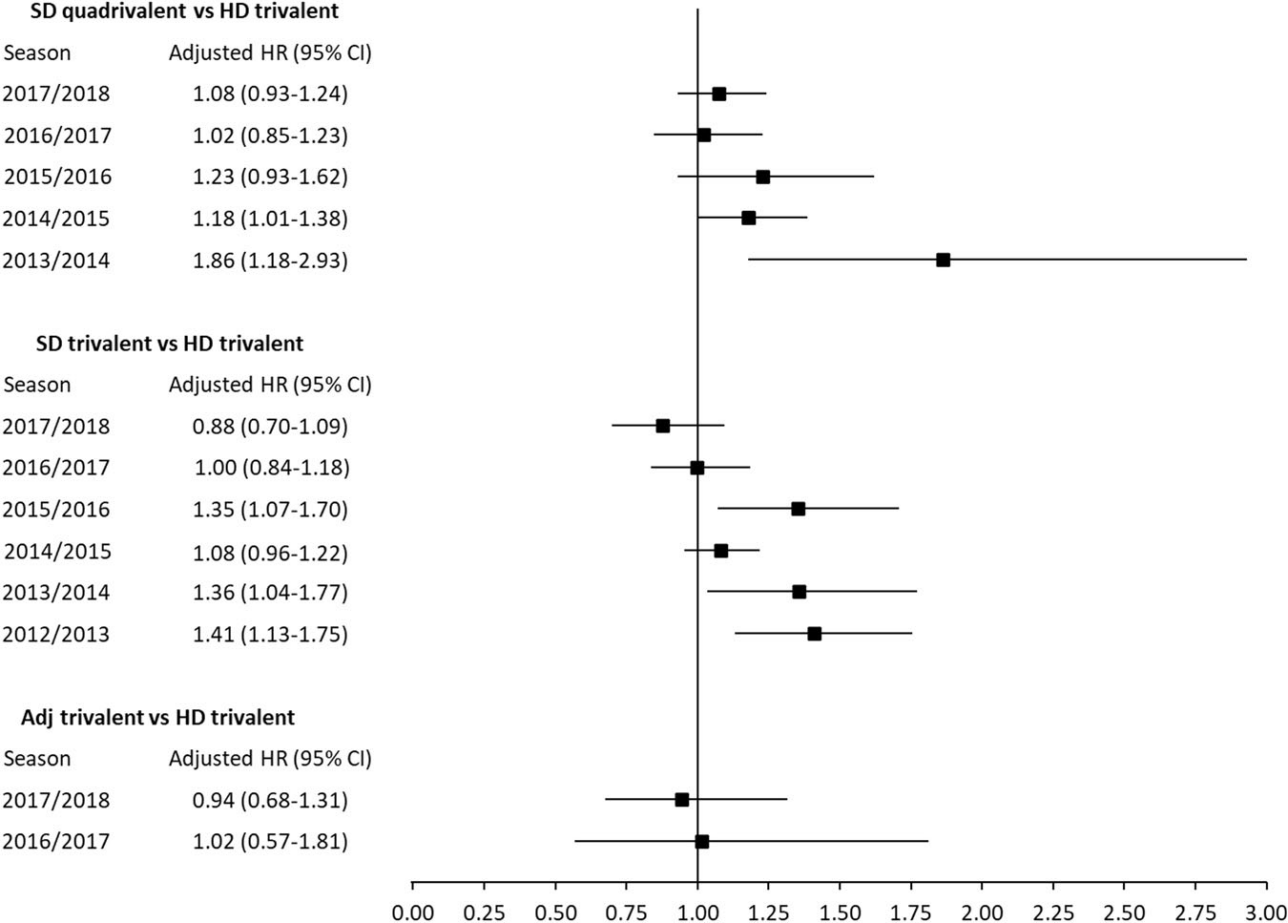
Adjusted hazard ratios (HRs) for influenza (hospitalization/emergency room visit). The adjusted HR point estimates and 95% confidence intervals are presented for each comparison and each influenza season. The high-dose trivalent vaccine is used as the reference group.

**Fig. 2:**
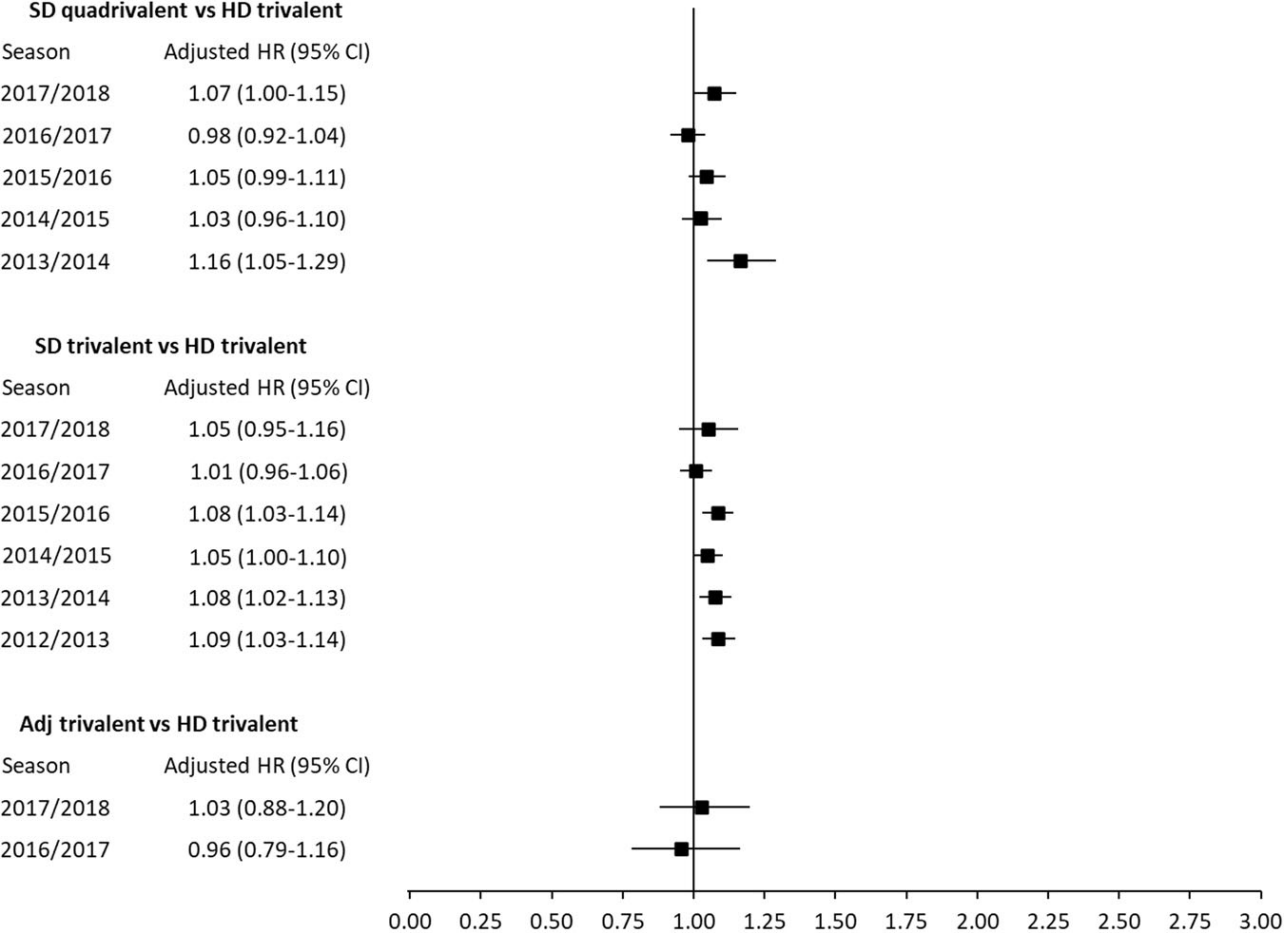
Adjusted hazard ratios (HRs) for pneumonia (hospitalization/emergency room visit). The adjusted HR point estimates and 95% confidence intervals are presented for each comparison and each influenza season. The high-dose trivalent vaccine is used as the reference group.

The pooled analyses did not reveal statistically significant heterogeneity between relative risk estimates of different vaccines across seasons for any outcome (*p* > 0.05 for overall LRT of season × vaccine interactions). In separate analyses per season, some outcome rates differed across subjects who used different vaccines (*p* < 0.05 for overall LRT). Figures 1–2 show, for each outcome, the season-specific aHRs, with 95% CI, for each of the three vaccines relative to HD-trivalent. Compared to HD-trivalent, SD-quadrivalent was associated with a higher risk for both outcomes in 2013/2014 (Figs. 1 and 2). Compared to HD-trivalent, subjects who received SD-trivalent had a higher risk of influenza-related hospitalization/ER visit (Fig. 1) and pneumonia-related hospitalization/ER visit (Fig. 2) in 2015/2016, 2013/2014, and 2012/2013 seasons. Table 3 reports the results of the pairwise comparisons not involving HD-trivalent, based on multivariable analyses in each season, which did not indicate statistically significant association with outcome risks. For each season, the proportional hazards hypothesis was not rejected for any vaccine, indicating that the relative effectiveness of alternative vaccine remained approximately constant during a year after vaccination^12^.

**Table 3:** Adjusted hazard ratios and 95% confidence intervals for outcomes in pairwise comparisons (reference groups are indicated in the columns).

Outcome/influenza season	Adjuvanted trivalent	Standard-dose trivalent
Influenza^a^: standard-dose quadrivalent		
2017/2018	1.14 (0.81–1.60)	1.23 (0.97–1.54)
2016/2017	1.01 (0.57–1.81)	1.03 (0.84–1.26)
2015/2016	NA	0.91 (0.70–1.18)
2014/2015	NA	1.09 (0.95–1.26)
2013/2014	NA	1.37 (0.92–2.04)
Pneumonia^a^: standard-dose quadrivalent		
2017/2018	1.04 (0.89–1.22)	1.02 (0.92–1.13)
2016/2017	1.03 (0.84–1.25)	0.97 (0.91–1.04)
2015/2016	NA	0.96 (0.91–1.02)
2014/2015	NA	0.98 (0.92–1.04)
2013/2014	NA	1.08 (0.99–1.19)
Influenza^a^: standard-dose trivalent		
2017/2018	0.93 (0.64–1.36)	NA
2016/2017	0.98 (0.55–1.76)	NA
Pneumonia^a^: standard-dose trivalent		
2017/2018	1.03 (0.87–1.21)	NA
2016/2017	1.06 (0.87–1.29)	NA

^a^Hospitalization/emergency room visits.

### Secondary analyses

The adjusted pooled relative vaccine effectiveness (RVE) analyses showed that SD-quadrivalent was 15.6% (95% CI 25.8–6.2) less effective than HD-trivalent to prevent influenza-related hospitalization/ER visit. The secondary analyses corroborate the above results and are presented in Supplementary Table 10.

## Discussion

In persons aged ≥65 years, across six influenza seasons, both SD-trivalent and SD-quadrivalent were associated with a higher risk of hospitalized/ER visit influenza and hospitalized/ER visit pneumonia compared with HD-trivalent. We did not clearly demonstrate risk differences between SD vaccine formulations and between adjuvanted trivalent and other vaccines.

Our study confirms prior findings of greater effectiveness of HD-trivalent compared to SD-trivalent in preventing medical encounters for influenza or pneumonia in seniors from 2012/2013 through 2018/2019 season^6–9,13–16^. However, previous studies found similar rates of influenza and influenza-related complications among seniors vaccinated with HD-trivalent or SD-trivalent in some seasons^6,8,9,15,17^. Our results differ from previous cohort studies from the United States, Canada, and Italy, which have demonstrated higher effectiveness of adjuvanted trivalent or cell-cultured quadrivalent vaccines compared with SD-trivalent in seniors^16,18–21^. This disagreement may result from different aspects of the studies, such as the base populations (especially in the Canadian and Italian investigations), outcome definitions, or influenza seasons (two of the aforementioned studies considered 2006/2007–2011/2012 seasons). In the literature, comparative studies on HD-trivalent vs. SD-quadrivalent are still scarce, especially in real-world settings. Two case–control studies showed that across the 2015/2016 and 2018/2019 seasons, and compared with a group exposed to either SD-trivalent or SD-quadrivalent, seniors vaccinated with HD-trivalent had marginally significant fewer cases of laboratory-confirmed influenza. Investigators were unable to show differences among groups in each separate season, which could be due to insufficient sample size^10,11^. A recent randomized controlled trial (RCT) in patients with high-risk cardiovascular disease found that HD-trivalent did not reduce the risk for all-cause mortality or hospitalization for cardiac or pulmonary events compared with SD-quadrivalent vaccine across three seasons (2016/2017–2018/2019)^22^. In our study of real-world data, we also did not detect differences for risk of hospitalized influenza and pneumonia between HD-trivalent and SD-quadrivalent in the 2016/2017 and 2017/2018 seasons. The results of our pooled analyses, which showed lower risk associated with HD-trivalent, were driven by the 2014/2015 and 2013/2014 seasons. In addition, this RCT is not directly comparable to our study, because it did not exclusively include seniors (~40% were <65 years).

Some differences in vaccine-specific risks observed across seasons are potentially explained by the variation of the influenza season’s severity and the match between vaccines and circulating virus strains. The latter was demonstrated by the fluctuation of vaccine effectiveness in the US population: during our study period, vaccine effectiveness in seniors (combined across all vaccine types) was the highest in 2013/2014 (50%, 95% CI 16 to 71) and the lowest in 2017/2018 (17%, 95% CI −14 to 39)^23,24^. In the United States, 2017/2018 was considered the most severe season since the 2009 H1N1 pandemic and rates of hospitalization due to influenza in all age groups were higher than in previous years^25^. In our study, in the 2016/2017 and 2017/2018 seasons, we could not establish differences for risk of hospitalized influenza and pneumonia associated with different vaccines. Our results are consistent with earlier cohort studies in the United States that have shown relative vaccine effectiveness variations across seasons^6,8,9^.

Influenza immunization is an important and effective strategy to mitigate the burden of influenza infections on individuals and healthcare systems, especially among older adults amid the COVID-19 pandemic. Our findings provide relevant and timing evidence of comparative effectiveness among four types of influenza vaccines in seniors and may support policy-makers to recommend, when available, HD-trivalent over other vaccines in seniors.

One potential limitation of our study is the use of hospital ICD codes to define influenza and pneumonia cases, which would likely capture only severe cases. In addition, we could not determine vaccine effectiveness across different influenza types and subtypes. Another potential limitation is confounding by indication (groups at higher or lower risk may be preferentially given a specific vaccine type). We attempted to minimize this by adjusting for an extensive set of patient characteristics, including indicators of frailty health, comorbidities, region of residence, and health plan type. As always, some residual unmeasured confounding by indication could remain. However, the CDC does not recommend specific vaccine types based upon patient characteristics and we specifically chose our design to include a broad spectrum of seniors who had all been vaccinated with one type of vaccine or another.

As influenza vaccine effectiveness varies each year (as discussed above), calendar-year issues could potentially introduce confounding into pooled analyses if certain types of vaccines are used more in one given year vs. another; e.g., SD-trivalent vaccines were less used during 2016–2018 after newer vaccines were approved for seniors. Our analyses stratified by seasons overcome the issue of calendar-year effects, although calendar effects could arise in pooled analyses. We did consider this for SD-trivalent vaccine and we excluded from the pooled analyses this vaccine in the 2016/2017 and 2017/2018 seasons.

On the other hand, our study has some important strengths. First, we analyzed relative vaccine effectiveness throughout six influenza seasons. Moreover, we included a large number of vaccinated seniors, which ensured sufficient statistical power and precision to compare serious events, such as hospitalization for influenza and pneumonia, among four different influenza vaccines. However, as the adjuvanted trivalent vaccine was only recently available, some corresponding confidence intervals are relatively wide. Finally, we applied two methods to estimate comparative effects, one using a time-to-event approach with aHRs and another using rate ratio to calculate a measure of relative vaccine effectiveness.

In conclusion, persons aged ≥65 years, who received HD-trivalent vaccine, were less likely to be hospitalized or admitted to an emergency department for influenza or pneumonia than recipients of SD-trivalent or SD-quadrivalent. The comparisons of adjuvanted trivalent with HD-trivalent or SD-quadrivalent did not indicate differences with respect to both outcomes, although adjuvanted vaccine had a small sample size, which limits the precision of our estimates. We did not clearly demonstrate differences in outcomes between SD-quadrivalent and SD-trivalent.

## Methods

### Data source

We used data from the IBM MarketScan Medicare Supplemental Databases from 1 January 2011 to 31 December 2018. These databases include administrative health information on physician office visits and services (including vaccination), ER visits, hospital stays, drug prescriptions filled, and health insurance enrollment data. In MarketScan Medicare databases, there are almost 1.1 million individuals aged 65 years or older (2018), which represents 2% of the 53 million US senior residents (2019 census, https://www.census.gov/topics/population/age-and-sex/data/tables).

### Study population

We defined six study years that began on 1 September and ended on 31 August of the subsequent year, covering six influenza seasons in the Northern Hemisphere (2012/2013–2017/2018 seasons). For each study year, we selected seniors (65 years of age or older) who received SD-trivalent, HD-trivalent, SD-quadrivalent, or adjuvanted trivalent vaccines between 1 September and 15 August. Time zero was defined as 14 days after the vaccination date to allow for the development of vaccine-induced immunity. We required that individuals had continuous enrollment in their medical and pharmacy insurance plans for 12 months before the vaccination date, to have baseline information on comorbidity and other factors. We excluded individuals residing in nursing homes or receiving hospice care on the vaccination date, because their medical encounters may not be reliably captured. Those who had an influenza diagnosis before the time zero within each study year were also excluded. Subjects could be included in more than one influenza season.

### Influenza vaccine exposure

We identified influenza vaccinations in outpatient claims using Current Procedural Terminology codes: 90654–90661, 90724, and Q2034–Q2038 for SD-trivalent; 90662 for HD-trivalent; 90630, 90674, 90685–90688, 90694, and 90756 for SD-quadrivalent; and 90653 adjuvanted trivalent. In our study, 90% of the immunization procedures took place in outpatient offices, 5% in Mass Immunization Centers (it may be public health center, pharmacy, or physician office), and 0.1% in pharmacies.

### Outcomes

Our primary outcomes were as follows: (1) hospitalization or ER visit with a principal or secondary diagnosis of influenza (ICD-9 codes 487-488 or ICD-10 J09-J11) and (2) hospitalization or ER visit with a principal or secondary diagnosis of pneumonia (ICD-9 480-486 or ICD-10 J12-J18). Pneumonia is a possible influenza-related complication.

### Covariates

Baseline covariates measured at the vaccination date included the following: age, sex, time span between 1 September in each year and the date of vaccination (in days), health plan type (Comprehensive, Health Maintenance Organization, Preferred Provider Organization, Other), employment status (retiree, active full time, spouse/dependent, other/unknown), region of residence (Northeast, North Central, South, West), and area of residence (urban, rural). We also adjusted for potential confounders measured during the year prior to vaccination: hospitalization for pneumonia, Charlson Comorbidity Index (as well as specific ICD-based indicators of diabetes, chronic obstructive pulmonary disease/asthma, myocardial infarction, congestive heart failure, peripheral vascular disease, renal disease, and malignancy), frail physical health (based on indicators of home hospital bed, wheelchair, home oxygen, ambulance/life support)^26^, use of bronchodilator inhalers, use of inhaled or oral corticosteroids, number of ER visits, and number of previous physician visits.

### Statistical analysis

Descriptive statistics were used to describe recipients of different vaccines in each influenza season. For each of the two outcomes and each of the six influenza seasons, we estimated crude incidence rates per 10,000 person-weeks with 95% CIs for patients who received each vaccine.

Main analyses compared the effectiveness of influenza vaccines, separately for each outcome, and relied on multivariable Cox proportional hazards models. The analyses were carried out for data pooled from all six seasons and repeated separately for each season. We excluded from the pooled analyses the SD-trivalent vaccine in the 2016/2017 and 2017/2018 seasons due to potential bias associated with calendar-year effects. In the pooled analyses, statistical inference relied on robust sandwich SEs to account for clustering due to repeated observations of the same patients in different years. To assess if the relative effectiveness of alternative vaccines differed across the seasons, we included a series of two-way vaccine-by-season interactions and tested their joint statistical significance using the overall LRT, at *α* = 0.05.

Individual time-to-event was defined as the time elapsed between 14 days after the patient’s vaccination date and the earliest occurrence of the event of interest. Patients who had no event until the earliest of (1) end of healthcare insurance coverage, (2) admission to a nursing home or hospice facility, (3) administration of a subsequent influenza vaccine in the same study year, (4) death (only in-hospital death information was available), and (5) end of the study year on 31 August were censored at that time. Each model included all variables listed in the previous sub-section as covariates. In addition, for the pneumonia outcome, we adjusted for a binary indicator of pneumococcal vaccine at any time before the influenza vaccination date. In the pooled analyses, we also adjusted for the influenza season. Results for each vaccine were summarized by aHRs, with 95% CI, relative to the reference category of HD-trivalent vaccine. The overall LRT with 3 or 2 degrees of freedom (depending on whether in a given season 3 or 4 vaccines were available), at *α* = 0.05, was used to assess whether there were statistically significant differences between adjusted hazard rates for recipients of different influenza vaccines. If LRT rejected the null hypothesis of no differences between vaccines, statistically significant results for pairwise comparisons of each vaccine pair were identified based on the covariance matrix of all estimated regression coefficients and the corresponding aHR with 95% CI’s were reported. Proportional hazards assumption was tested using a non-parametric residual-based method^27^, separately for each season and each vaccine.

In secondary analyses, we estimated multivariable Poisson regression models with the same covariates as in the Cox models. Then, based on the resulting adjusted rate ratios, we estimated RVE = (1 − adjusted rate ratio) × 100, using the HD-trivalent vaccine as the reference.

All analyses were performed using SAS version 9.4 (SAS Institute, Cary, NC).

### Ethics approval

The study was approved by the Research Ethics Office of the Faculty of Medicine, McGill University (IRB Study Number A04-M47-12B). Informed consent is not applicable, because the study used de-identified claims data.

### Reporting summary

Further information on research design is available in the Nature Research Reporting Summary linked to this article.

## Supplementary information

Supplementary Information

Reporting Summary

## Data Availability

IBM MarketScan Medicare Supplemental Database is not in the public domain but is available to researchers at a cost. Data were used in compliance with privacy and confidentiality requirements.
