# Supplementary material for: Relative effectiveness of influenza vaccines in elderly persons in the United States, 2012/2013-2017/2018 seasons
Source: NPJ Vaccines. 2021 Aug 24;6:108. doi: 10.1038/s41541-021-00373-w (PMC8385076; doi:10.1038/s41541-021-00373-w)
Supplement: Supplementary file 1 — Supplementary Information [file 41541_2021_373_MOESM1_ESM.pdf]

Supplementary Information

Supplementary Table 1. Reasons for exclusion of subjects in each influenza season.

Supplementary Table 2. Baseline characteristics of included subjects in the 2017/2018 season (N=211,536).

Supplementary Table 3. Baseline characteristics of included subjects in the 2016/2017 season (N=368,291).

Supplementary Table 4. Baseline characteristics of included subjects in the 2015/2016 season (N= 359,188).

Supplementary Table 5. Baseline characteristics of included subjects in the 2014/2015 season (N=417,885).

Supplementary Table 6. Baseline characteristics of included subjects in the 2013/2014 season (N=484,341).

Supplementary Table 7. Baseline characteristics of included subjects in the 2012/2013 season (N=533,616).

Supplementary Table 8. Incidence rates per 10,000 person-weeks (p-w) and 95% confidence intervals (CI) for outcomes per influenza season and vaccine type.

Supplementary Table 9. Adjusted hazard ratios (HR) and 95% confidence intervals (CI) for outcomes in the pooled analyses (six seasons 2012/2013-2017/2018).

Supplementary Table 10. Adjusted relative vaccine effectiveness (RVE) and 95% confidence intervals (CI) for outcomes, using the high-dose trivalent vaccine as the reference group.

Supplementary Table 1. Reasons for exclusion of subjects in each influenza season.

	Influenza season					
	**2017/2018**	**2016/2017**	**2015/2016**	**2014/2015**	**2013/2014**	**2012/2013**
Individuals who received influenza vaccination between September 1 and August 15	274,885	439,409	453,007	693,052	771,514	862,612
Excluded individuals						
Age < 65 years old*	10,864	17,235	8,775	8,927	10,492	11,423
< 1 year of continuous enrollment in insurance plan**	52,028	53,095	84,125	265,173	275,576	315,843
Residing in nursing homes or receiving hospice care*	123	436	577	678	700	1,121
Influenza diagnosis before observation period	334	352	342	389	405	609
Final selected cohort	211,536	368,291	359,188	417,885	484,341	533,616

* At vaccination date. ** Before vaccination date.

Supplementary Table 2. Baseline characteristics of included subjects in the 2017/2018 season (N=211,536).

Characteristics	Adjuvanted Trivalent (N=9,061, 4.3%)	High-dose Trivalent (N=113,895, 53.8%)	Standard-dose Trivalent (N=23,965, 11.3%)	Quadrivalent (N=64,615, 30.6%)
Sex (Male)	4069 (44.9%)	51595 (45.3%)	11040 (46.1%)	29399 (45.5%)
Age (median, interquartile range)	74 (69-81)	74 (69-80)	74 (69-81)	74 (69-80)
Hospitalization for pneumonia *	238 (2.6%)	2853 (2.5%)	640 (2.7%)	1486 (2.3%)
Charlson Comorbidity Index *				
Mean (standard deviation)	1.8 (2.0)	1.8 (2.1)	2.0 (2.1)	1.8 (2.0)
Median (interquartile range)	1.0 (0.0-3.0)	1.0 (0.0-3.0)	1.0 (0.0-3.0)	1.0 (0.0-3.0)
Comorbidities*				
Diabetes	2960 (32.7%)	35337 (31%)	8130 (33.9%)	19915 (30.8%)
COPD	1678 (18.5%)	21908 (19.2%)	5228 (21.8%)	12563 (19.4%)
Cancer	1212 (13.4%)	15717 (13.8%)	3414 (14.3%)	8495 (13.2%)
Cerebrovascular disease	1061 (11.7%)	13616 (12%)	3533 (14.7%)	7617 (11.8%)
Congestive heart failure	1034 (11.4%)	12895 (11.3%)	3093 (12.9%)	7338 (11.4%)
Asthma	585 (6.5%)	7646 (6.7%)	1687 (7%)	4384 (6.8%)
Myocardial infarction	266 (2.9%)	3722 (3.3%)	842 (3.5%)	2059 (3.2%)
Peripheral vascular disease	1008 (11.1%)	12835 (11.3%)	3316 (13.8%)	7384 (11.4%)
Rheumatoid arthritis	257 (2.8%)	2758 (2.4%)	686 (2.9%)	1680 (2.6%)
Inflammatory bowel disease	90 (1%)	1160 (1%)	230 (1%)	604 (0.9%)
Frail physical health ¥	1018 (11.2%)	13843 (12.2%)	2942 (12.3%)	7694 (11.9%)
Past use of drugs *				
Bronchodilators	737 (8.1%)	9090 (8%)	1992 (8.3%)	5024 (7.8%)
Corticosteroids	2359 (26%)	29256 (25.7%)	6222 (26%)	16350 (25.3%)
Anticholinergics	448 (4.9%)	5692 (5%)	1157 (4.8%)	3023 (4.7%)
DMARDs and biologic agents	290 (3.2%)	3449 (3%)	751 (3.1%)	1989 (3.1%)
Period of vaccination ±				
Pre-influenza season	2165 (23.9%)	34337 (30.2%)	5664 (23.6%)	17273 (26.7%)
Influenza season	6894 (76.1%)	79496 (69.8%)	18296 (76.3%)	47334 (73.3%)
Post-influenza season	2 (0%)	62 (0.1%)	5 (0%)	8 (0%)
Health plan type €				
Comprehensive	3951 (44%)	42081 (37.9%)	9509 (39.8%)	20404 (33.9%)
HMO	1209 (13.5%)	20065 (18.1%)	2405 (10.1%)	10220 (17%)
PPO	3163 (35.2%)	41062 (36.9%)	9525 (39.9%)	24539 (40.8%)
Other	652 (7.3%)	7981 (7.2%)	2449 (10.3%)	5009 (8.3%)
Employment status				
Retiree	974 (10.8%)	13309 (11.7%)	2273 (9.5%)	7818 (12.1%)
Other/Unknown	1904 (21%)	23194 (20.4%)	4647 (19.4%)	16281 (25.2%)
Active Full Time	5339 (58.9%)	67410 (59.2%)	14717 (61.4%)	35498 (54.9%)
Spouse/dependent	844 (9.3%)	9982 (8.8%)	2328 (9.7%)	5018 (7.8%)
Urban area	8409 (92.8%)	102739 (90.2%)	20429 (85.3%)	57110 (88.4%)
Region £				
Northeast	2205 (24.4%)	31933 (28.1%)	7827 (32.7%)	18773 (29.1%)

Characteristics	Adjuvanted Trivalent (N=9,061, 4.3%)	High-dose Trivalent (N=113,895, 53.8%)	Standard-dose Trivalent (N=23,965, 11.3%)	Quadrivalent (N=64,615, 30.6%)
North Central	2689 (29.7%)	34690 (30.5%)	6051 (25.3%)	16788 (26%)
South	2596 (28.7%)	33119 (29.1%)	7709 (32.2%)	21447 (33.2%)
West	1554 (17.2%)	13933 (12.3%)	2365 (9.9%)	7551 (11.7%)

*12 months before vaccination date. ¥ Frail physical health is a composite indicator that includes home hospital bed, wheelchair, home oxygen, ambulance/life support. € 7,312 observations were missing for health plan type. £ 306 observations were missing for region. COPD: chronic obstructive pulmonary disease. HMO: Health Maintenance Organization. PPO: Preferred Provider Organization. ± Pre-influenza season: Sept 1/Sept 30; influenza season: Oct 1/May 19; post-influenza season: May 20/Aug 31. Periods were defined according to seasonal surveillance reports from the Centers for Disease Control and Prevention (https://www.cdc.gov/flu/season/past-flu-seasons.htm).

Supplementary Table 3. Baseline characteristics of included subjects in the 2016/2017 season (N=368,291).

Characteristics	Adjuvanted Trivalent (N=4,480, 1.2%)	High-dose Trivalent (N=182,786, 49.6%)	Standard-dose Trivalent (N=99,217, 26.9%)	Quadrivalent (N=81,808, 22.2%)
Sex (Male)	1976 (44.1%)	80609 (44.1%)	44551 (44.9%)	36670 (44.8%)
Age (median, interquartile range)	74 (69-81)	74 (69-81)	74 (69-81)	73 (69-80)
Hospitalization for pneumonia *	99 (2.2%)	3607 (2%)	2572 (2.6%)	1747 (2.1%)
Charlson Comorbidity Index *				
Mean (standard deviation)	1.7 (2.0)	1.6 (1.9)	1.9 (2.0)	1.6 (1.9)
Median (interquartile range)	1.0 (0.0-3.0)	1.0 (0.0-2.0)	1.0 (0.0-3.0)	1.0 (0.0-3.0)
Comorbidities*				
Diabetes	1345 (30%)	50364 (27.6%)	31966 (32.2%)	22881 (28%)
COPD	794 (17.7%)	30675 (16.8%)	20788 (21%)	14095 (17.2%)
Cancer	579 (12.9%)	23384 (12.8%)	13507 (13.6%)	10341 (12.6%)
Cerebrovascular disease	450 (10%)	19348 (10.6%)	13425 (13.5%)	8406 (10.3%)
Congestive heart failure	459 (10.3%)	17429 (9.5%)	12356 (12.5%)	8139 (10%)
Asthma	280 (6.3%)	11076 (6.1%)	7037 (7.1%)	5045 (6.2%)
Myocardial infarction	129 (2.9%)	5212 (2.9%)	3388 (3.4%)	2521 (3.1%)
Peripheral vascular disease	425 (9.5%)	17804 (9.7%)	12505 (12.6%)	7861 (9.6%)
Rheumatoid arthritis	132 (3%)	4344 (2.4%)	2891 (2.9%)	2025 (2.5%)
Inflammatory bowel disease	41 (0.9%)	1800 (1%)	971 (1%)	769 (0.9%)
Frail physical health [¥]	618 (13.8%)	23381 (12.8%)	14982 (15.1%)	11064 (13.5%)
Past use of drugs *				
Bronchodilators	335 (7.5%)	11942 (6.5%)	7297 (7.4%)	5482 (6.7%)
Corticosteroids	975 (21.8%)	35104 (19.2%)	21576 (21.8%)	16830 (20.6%)
Anticholinergics	245 (5.5%)	7841 (4.3%)	4558 (4.6%)	3485 (4.3%)
DMARDs and biologic agents	152 (3.4%)	4920 (2.7%)	2818 (2.8%)	2396 (2.9%)
Period of vaccination				
Pre-influenza season	1259 (28.1%)	52958 (29%)	30766 (31%)	20239 (24.7%)
Influenza season	3208 (71.6%)	129656 (70.9%)	68424 (69%)	61506 (75.2%)
Post-influenza season	13 (0.3%)	172 (0.1%)	27 (0%)	63 (0.1%)
Health plan type [€]				
Comprehensive	1328 (29.7%)	49263 (27%)	33611 (33.9%)	21135 (26%)
HMO	783 (17.5%)	25866 (14.2%)	8025 (8.1%)	15899 (19.6%)
PPO	1941 (43.4%)	92936 (51%)	48948 (49.4%)	37876 (46.6%)
Other	422 (9.4%)	14246 (7.8%)	8484 (8.6%)	6369 (7.8%)
Employment status				
Retiree	254 (5.7%)	15197 (8.3%)	7531 (7.6%)	8175 (10%)
Other/Unknown	1172 (26.2%)	23761 (13%)	11637 (11.7%)	14699 (18%)
Active Full Time	2754 (61.5%)	130687 (71.5%)	71174 (71.7%)	53504 (65.4%)
Spouse/dependent	300 (6.7%)	13141 (7.2%)	8875 (9%)	5430 (6.6%)
Urban area	4032 (90%)	158452 (86.7%)	85278 (86%)	67549 (82.6%)
Region [£]				
Northeast	866 (19.3%)	34101 (18.7%)	23497 (23.7%)	17612 (21.5%)

Characteristics	Adjuvanted Trivalent (N=4,480, 1.2%)	High-dose Trivalent (N=182,786, 49.6%)	Standard-dose Trivalent (N=99,217, 26.9%)	Quadrivalent (N=81,808, 22.2%)
North Central	1536 (34.3%)	85559 (46.8%)	36835 (37.2%)	32790 (40.1%)
South	1205 (26.9%)	45627 (25%)	28882 (29.1%)	22528 (27.6%)
West	872 (19.5%)	17429 (9.5%)	9946 (10%)	8840 (10.8%)

*12 months before vaccination date. ¥ Frail physical health is a composite indicator that includes home hospital bed, wheelchair, home oxygen, ambulance/life support. € 1,159 observations were missing for health plan type. £ 166 observations were missing for region. COPD: chronic obstructive pulmonary disease. HMO: Health Maintenance Organization. PPO: Preferred Provider Organization. ± Pre-influenza season: Sept 1/ Oct 1; influenza season: Oct 2/May 20; post-influenza season: May 21/Aug 31. Periods were defined according to seasonal surveillance reports from the Centers for Disease Control and Prevention (https://www.cdc.gov/flu/season/past-flu-seasons.htm).

Supplementary Table 4. Baseline characteristics of included subjects in the 2015/2016 season (N= 359,188).

Characteristics	High-dose Trivalent (N=140,535, 39.1%)	Standard-dose Trivalent (N=142,075, 39.6%)	Quadrivalent (N=76,578, 21.3%)
Sex (Male)	62248 (44.3%)	63600 (44.8%)	34527 (45.1%)
Age (median, interquartile range)	74 (69-81)	75 (69-81) (%)	74 (69-80) (%)
Hospitalization for pneumonia *	2827 (2%)	3836 (2.7%)	1780 (2.3%)
Charlson Comorbidity Index *			
Mean (standard deviation)	1.4 (1.7)	1.7 (1.9)	1.4 (1.7)
Median (interquartile range)	1.0 (0.0-2.0)	1.0 (0.0-3.0)	1.0 (0.0-2.0)
Comorbidities*			
Diabetes	37351 (26.6%)	43963 (30.9%)	20714 (27.1%)
COPD	23156 (16.5%)	28490 (20.1%)	13031 (17%)
Cancer	17475 (12.4%)	18946 (13.3%)	9664 (12.6%)
Cerebrovascular disease	14294 (10.2%)	18662 (13.1%)	7681 (10%)
Congestive heart failure	10383 (7.4%)	14270 (10%)	6043 (7.9%)
Asthma	8824 (6.3%)	10515 (7.4%)	4904 (6.4%)
Myocardial infarction	3851 (2.7%)	4774 (3.4%)	2135 (2.8%)
Peripheral vascular disease	8431 (6%)	11290 (8%)	4686 (6.1%)
Rheumatoid arthritis	4486 (3.2%)	5597 (3.9%)	2520 (3.3%)
Inflammatory bowel disease	1193 (0.9%)	1273 (0.9%)	625 (0.8%)
Frail physical health [¥]	17538 (12.5%)	22020 (15.5%)	10163 (13.3%)
Past use of drugs *			
Bronchodilators	10005 (7.1%)	10911 (7.7%)	5452 (7.1%)
Corticosteroids	27920 (19.9%)	30474 (21.5%)	16183 (21.1%)
Anticholinergics	6482 (4.6%)	7148 (5%)	3612 (4.7%)
DMARDs and biologic agents	3868 (2.8%)	3915 (2.8%)	2201 (2.9%)
Period of vaccination			
Pre-influenza season	39595 (28.2%)	45177 (31.8%)	18843 (24.6%)
Influenza season	100844 (71.8%)	96768 (68.1%)	57704 (75.4%)
Post-influenza season	96 (0.1%)	130 (0.1%)	31 (0%)
Health plan type [€]			
Comprehensive	35908 (25.6%)	46857 (33%)	19299 (25.3%)
HMO	21388 (15.3%)	16935 (11.9%)	17421 (22.8%)
PPO	74838 (53.4%)	71437 (50.4%)	35447 (46.5%)
Other	8110 (5.8%)	6640 (4.7%)	4146 (5.4%)
Employment status			
Retiree	12209 (8.7%)	10572 (7.4%)	7879 (10.3%)
Other/Unknown	16789 (12%)	15837 (11.2%)	13481 (17.6%)
Active Full Time	101538 (72.3%)	103082 (72.6%)	50180 (65.5%)
Spouse/dependent	9999 (7.1%)	12584 (8.9%)	5038 (6.6%)
Urban area	121885 (86.7%)	122113 (86%)	63913 (83.5%)
Region [£]			
Northeast	26785 (19.1%)	32360 (22.8%)	16824 (22%)

Characteristics	High-dose Trivalent (N=140,535, 39.1%)	Standard-dose Trivalent (N=142,075, 39.6%)	Quadrivalent (N=76,578, 21.3%)
North Central	69437 (49.4%)	60032 (42.3%)	31645 (41.3%)
South	31712 (22.6%)	34618 (24.4%)	19912 (26%)
West	12560 (8.9%)	15003 (10.6%)	8176 (10.7%)

*12 months before vaccination date. ¥ Frail physical health is a composite indicator that includes home hospital bed, wheelchair, home oxygen, ambulance/life support. € 762 observations were missing for health plan type. £ 124 observations were missing for region. COPD: chronic obstructive pulmonary disease. HMO: Health Maintenance Organization. PPO: Preferred Provider Organization. ± Pre-influenza season: Sept 1/ Oct 3; influenza season: Oct 4/May 21; post-influenza season: May 22/Aug 31. Periods were defined according to seasonal surveillance reports from the Centers for Disease Control and Prevention (https://www.cdc.gov/flu/season/past-flu-seasons.htm).

Supplementary Table 5. Baseline characteristics of included subjects in the 2014/2015 season (N=417,885).

Characteristics	High-dose Trivalent (N=121,953, 29.2%)	Standard-dose Trivalent (N=229,071, 54.8%)	Quadrivalent (N=66,861, 16.0%)
Sex (Male)	54562 (44.7%)	103360 (45.1%)	30277 (45.3%)
Age (median, interquartile range)	74 (69-80)	74 (69-81)	73 (69-80)
Hospitalization for pneumonia *	2327 (1.9%)	5234 (2.3%)	1230 (1.8%)
Charlson Comorbidity Index *			
Mean (standard deviation)	1.3 (1.6)	1.5 (1.7)	1.2 (1.6)
Median (interquartile range)	1.0 (0.0-2.0)	1.0 (0.0-2.0)	1.0 (0.0-2.0)
Comorbidities*			
Diabetes	31781 (26.1%)	66792 (29.2%)	17090 (25.6%)
COPD	18889 (15.5%)	41788 (18.2%)	10022 (15%)
Cancer	14685 (12%)	30029 (13.1%)	8165 (12.2%)
Cerebrovascular disease	12188 (10%)	27968 (12.2%)	6245 (9.3%)
Congestive heart failure	8133 (6.7%)	19858 (8.7%)	4477 (6.7%)
Asthma	7262 (6%)	15781 (6.9%)	3847 (5.8%)
Myocardial infarction	3080 (2.5%)	6832 (3%)	1758 (2.6%)
Peripheral vascular disease	6516 (5.3%)	15535 (6.8%)	3402 (5.1%)
Rheumatoid arthritis	3080 (2.5%)	6832 (3%)	1758 (2.6%)
Inflammatory bowel disease	1033 (0.9%)	2020 (0.9%)	530 (0.8%)
Frail physical health [¥]	15027 (12.3%)	31887 (13.9%)	8231 (12.3%)
Past use of drugs *			
Bronchodilators	8928 (7.3%)	17421 (7.6%)	4685 (7%)
Corticosteroids	26670 (21.9%)	50796 (22.2%)	14360 (21.5%)
Anticholinergics	5994 (4.9%)	11624 (5.1%)	3128 (4.7%)
DMARDs and biologic agents	3325 (2.7%)	6512 (2.8%)	1859 (2.8%)
Period of vaccination			
Pre-influenza season	31163 (25.6%)	52676 (23%)	12898 (19.3%)
Influenza season	90769 (74.4%)	176277 (77%)	53936 (80.7%)
Post-influenza season	21 (0%)	118 (0.1%)	27 (0%)
Health plan type [€]			
Comprehensive	33907 (27.9%)	71635 (31.3%)	16193 (24.3%)
HMO	18457 (15.2%)	30837 (13.5%)	15129 (22.7%)
PPO	62483 (51.3%)	117385 (51.3%)	31693 (47.5%)
Other	6887 (5.7%)	8953 (3.9%)	3691 (5.5%)
Employment status			
Retiree	9322 (7.6%)	15545 (6.8%)	6142 (9.2%)
Other/Unknown	26333 (21.6%)	51360 (22.4%)	17782 (26.6%)
Active Full Time	78297 (64.2%)	145400 (63.5%)	39046 (58.4%)
Spouse/dependent	8001 (6.6%)	16766 (7.3%)	3891 (5.8%)
Urban area	107542 (88.2%)	198047 (86.5%)	57339 (85.8%)
Region [£]			
Northeast	25652 (21.2%)	60666 (26.8%)	15731 (23.8%)

Characteristics	High-dose Trivalent (N=121,953, 29.2%)	Standard-dose Trivalent (N=229,071, 54.8%)	Quadrivalent (N=66,861, 16.0%)
North Central	53258 (44.1%)	87881 (38.8%)	27274 (41.3%)
South	30404 (25.2%)	53641 (23.7%)	15845 (24%)
West	11443 (9.5%)	24473 (10.8%)	7142 (10.8%)

*12 months before vaccination date. ¥ Frail physical health is a composite indicator that includes home hospital bed, wheelchair, home oxygen, ambulance/life support. € 635 observations were missing for health plan type. £ 4,476 observations were missing for region. COPD: chronic obstructive pulmonary disease. HMO: Health Maintenance Organization. PPO: Preferred Provider Organization. ± Pre-influenza season: Sept 1/ Sept 27; influenza season: Sept 28/May 23; post-influenza season: May 24/Aug 31. Periods were defined according to seasonal surveillance reports from the Centers for Disease Control and Prevention (https://www.cdc.gov/flu/season/past-flu-seasons.htm).

Supplementary Table 6. Baseline characteristics of included subjects in the 2013/2014 season (N=484,341).

Characteristics	High-dose Trivalent (N=93,178, 19.2%)	Standard-dose Trivalent (N=369,326, 76.2%)	Quadrivalent (N=21,837, 4.5%)
Sex (Male)	41486 (44.5%)	164831 (44.6%)	9989 (45.7%)
Age (median, interquartile range)	74 (69-81)	74 (69-81)	
Hospitalization for pneumonia *	1972 (2.1%)	8870 (2.4%)	457 (2.1%)
Charlson Comorbidity Index *			
Mean (standard deviation)	1.2 (1.6)	1.4 (1.7)	
Median (interquartile range)	1.0 (0.0-2.0)	1.0 (0.0-2.0)	
Comorbidities*			
Diabetes	24385 (26.2%)	101642 (27.5%)	5201 (23.8%)
COPD	14547 (15.6%)	64358 (17.4%)	2993 (13.7%)
Cancer	11363 (12.2%)	46723 (12.7%)	2594 (11.9%)
Cerebrovascular disease	9429 (10.1%)	41409 (11.2%)	1938 (8.9%)
Congestive heart failure	6213 (6.7%)	29118 (7.9%)	1477 (6.8%)
Asthma	5381 (5.8%)	23960 (6.5%)	1164 (5.3%)
Myocardial infarction	2241 (2.4%)	11050 (3%)	544 (2.5%)
Peripheral vascular disease	4723 (5.1%)	22100 (6%)	991 (4.5%)
Rheumatoid arthritis	2241 (2.4%)	11050 (3%)	544 (2.5%)
Inflammatory bowel disease	753 (0.8%)	3088 (0.8%)	152 (0.7%)
Frail physical health [¥]	12028 (12.9%)	50648 (13.7%)	2624 (12%)
Past use of drugs *			
Bronchodilators	7199 (7.7%)	28421 (7.7%)	1514 (6.9%)
Corticosteroids	20679 (22.2%)	82908 (22.5%)	4741 (21.7%)
Anticholinergics	4792 (5.1%)	19216 (5.2%)	1043 (4.8%)
DMARDs and biologic agents	2418 (2.6%)	9926 (2.7%)	589 (2.7%)
Period of vaccination			
Pre-influenza season	26394 (28.3%)	101928 (27.6%)	3311 (15.2%)
Influenza season	66676 (71.6%)	266839 (72.3%)	18481 (84.6%)
Post-influenza season	108 (0.1%)	559 (0.2%)	45 (0.2%)
Health plan type [€]			
Comprehensive	28479 (30.7%)	113695 (30.9%)	5080 (23.3%)
HMO	15641 (16.9%)	73648 (20%)	6573 (30.2%)
PPO	44574 (48%)	170378 (46.2%)	9144 (42%)
Other	4144 (4.5%)	10833 (2.9%)	967 (4.4%)
Employment status			
Retiree	5124 (5.5%)	20010 (5.4%)	1854 (8.5%)
Other/Unknown	19530 (21%)	89363 (24.2%)	6064 (27.8%)
Active Full Time	62361 (66.9%)	235324 (63.7%)	12840 (58.8%)
Spouse/dependent	6163 (6.6%)	24629 (6.7%)	1079 (4.9%)
Urban area	80402 (86.3%)	317457 (86%)	19505 (89.3%)
Region [£]			
Northeast	21234 (22.9%)	101838 (27.7%)	3846 (17.7%)

Characteristics	High-dose Trivalent (N=93,178, 19.2%)	Standard-dose Trivalent (N=369,326, 76.2%)	Quadrivalent (N=21,837, 4.5%)
North Central	35659 (38.5%)	126453 (34.4%)	9108 (41.9%)
South	23528 (25.4%)	74307 (20.2%)	5417 (24.9%)
West	12275 (13.2%)	64774 (17.6%)	3367 (15.5%)

*12 months before vaccination date. ¥ Frail physical health is a composite indicator that includes home hospital bed, wheelchair, home oxygen, ambulance/life support. € 1,185 observations were missing for health plan type. £ 2,536 observations were missing for region. COPD: chronic obstructive pulmonary disease. HMO: Health Maintenance Organization. PPO: Preferred Provider Organization. ± Pre-influenza season: Sept 1/ Sept 28; influenza season: Sept 29/May 17; post-influenza season: May 18/Aug 31. Periods were defined according to seasonal surveillance reports from the Centers for Disease Control and Prevention (https://www.cdc.gov/flu/season/past-flu-seasons.htm).

Supplementary Table 7. Baseline characteristics of included subjects in the 2012/2013 season (N=533,616).

Characteristics	High-dose Trivalent (N=75,876, 14.2%)	Standard-dose Trivalent (N=457,740, 85.8%)
Sex (Male)	33437 (44.1%)	201793 (44.1%)
Age (median, interquartile range)	74 (69-81) (%)	74 (69-81) (%)
Hospitalization for pneumonia *	1481 (2%)	9631 (2.1%)
Charlson Comorbidity Index *		
Mean (standard deviation)	1.1 (1.5) (%)	1.2 (1.6) (%)
Median (interquartile range)	1.0 (0.0-2.0) (%)	1.0 (0.0-2.0) (%)
Comorbidities*		
Diabetes	19031 (25.1%)	113944 (24.9%)
COPD	10829 (14.3%)	69716 (15.2%)
Cancer	8441 (11.1%)	52177 (11.4%)
Cerebrovascular disease	7414 (9.8%)	47719 (10.4%)
Congestive heart failure	4921 (6.5%)	32346 (7.1%)
Asthma	3816 (5%)	25585 (5.6%)
Myocardial infarction	1775 (2.3%)	12519 (2.7%)
Peripheral vascular disease	3646 (4.8%)	24464 (5.3%)
Rheumatoid arthritis	1775 (2.3%)	12519 (2.7%)
Inflammatory bowel disease	557 (0.7%)	3395 (0.7%)
Frail physical health ¥	9749 (12.9%)	61280 (13.4%)
Past use of drugs *		
Bronchodilators	5846 (7.7%)	34230 (7.5%)
Corticosteroids	16291 (21.5%)	98631 (21.6%)
Anticholinergics	3964 (5.2%)	23336 (5.1%)
DMARDs and biologic agents	1943 (2.6%)	11871 (2.6%)
Period of vaccination		
Pre-influenza season	25806 (34%)	130615 (28.5%)
Influenza season	50048 (66%)	327041 (71.5%)
Post-influenza season	22 (0%)	84 (0%)
Health plan type €		
Comprehensive	23003 (30.6%)	126134 (27.7%)
HMO	12822 (17%)	99573 (21.9%)
PPO	36561 (48.6%)	216544 (47.6%)
Other	2853 (3.8%)	13052 (2.9%)
Employment status		
Retiree	2810 (3.7%)	21032 (4.6%)
Other/Unknown	19493 (25.7%)	127689 (27.9%)
Active Full Time	48659 (64.1%)	281764 (61.6%)
Spouse/dependent	4914 (6.5%)	27255 (6%)
Urban area	65245 (86%)	391154 (85.5%)
Region £		
Northeast	18880 (25%)	136446 (30%)
North Central	26125 (34.6%)	139185 (30.6%)

Characteristics	High-dose Trivalent (N=75,876, 14.2%)	Standard-dose Trivalent (N=457,740, 85.8%)
South	21620 (28.6%)	111100 (24.4%)
West	8891 (11.8%)	68777 (15.1%)

*12 months before vaccination date. ¥ Frail physical health is a composite indicator that includes home hospital bed, wheelchair, home oxygen, ambulance/life support. € 3,074 observations were missing for health plan type. £ 2,592 observations were missing for region. COPD: chronic obstructive pulmonary disease. HMO: Health Maintenance Organization. PPO: Preferred Provider Organization. ± Pre-influenza season: Sept 1/Sept 29; influenza season: Sept 30/May 18; post-influenza season: May 19/Aug 31. Periods were defined according to seasonal surveillance reports from the Centers for Disease Control and Prevention (https://www.cdc.gov/flu/season/past-flu-seasons.htm).

Supplementary Table 8. Incidence rates per 10,000 person-weeks (p-w) and 95% confidence intervals (CI) for outcomes per influenza season and vaccine type.

	Influenza (hospitalization/emergency room visit)			Pneumonia (hospitalization/emergency room visit)		
**Season/Vaccine type**	**Nº events**	**Person-weeks**	**Rate per 10,000 p-w (95% CI)**	**Nº events**	**Person-weeks**	**Rate per 10,000 p-w (95% CI**
**2017-2018**						
Standard-dose trivalent	97	686943	1.41 (1.16-1.72)	537	680816	7.89 (7.25-8.59)
Adjuvanted trivalent	38	255384	1.49 (1.08-2.05)	185	253158	7.31 (6.33-8.44)
High-dose trivalent	556	3478960	1.60 (1.47-1.74)	2480	3451707	7.18 (6.90-7.47)
Standard-dose quadrivalent	320	1931449	1.66 (1.49-1.85)	1390	1916441	7.25 (6.88-7.64)
**2016-2017**						
Standard-dose trivalent	234	3102172	0.75 (0.66-0.85)	2401	3067518	7.83 (7.52-8.15)
Adjuvanted trivalent	12	146803.7	0.82 (0.47-1.44)	104	145399	7.15 (5.90-8.67)
High-dose trivalent	354	5115792	0.69 (0.62-0.77)	3490	5066795	6.89 (6.67-7.12)
Standard-dose quadrivalent	174	2472890	0.70 (0.6-0.81)	1613	2450385	6.58 (6.27-6.91)
**2015-2016**						
Standard-dose trivalent	178	5684421	0.31 (0.27-0.36)	3947	5621117	7.02 (6.80-7.24)
High-dose trivalent	130	5721132	0.23 (0.19-0.27)	3100	5672243	5.47 (5.28-5.67)
Standard-dose quadrivalent	85	3044370	0.28 (0.23-0.35)	1763	3016798	5.84 (5.57-6.12)
**2014-2015**						
Standard-dose trivalent	843	7820756	1.08 (1.01-1.16)	5730	7746074	7.40 (7.21-7.59)
High-dose trivalent	413	4340688	0.95 (0.86-1.05)	2700	4307460	6.27 (6.04-6.51)
Standard-dose quadrivalent	244	2342737	1.04 (0.92-1.18)	1447	2325331	6.22 (5.91-6.55)
**2013-2014**						
Standard-dose trivalent	350	13486010	0.26 (0.23-0.29)	8435	13358755	6.31 (6.18-6.45)
High-dose trivalent	65	3523354	0.18 (0.14-0.23)	1937	3493044	5.55 (5.31-5.80)
Standard-dose quadrivalent	27	826939.4	0.33 (0.23-0.48)	487	819754.6	5.94 (5.44-6.49)
**2012-2013**						
Standard-dose trivalent	750	17249679	0.43 (0.40-0.46)	11048	17082177	6.47 (6.35-6.59)
High-dose trivalent	92	2913975	0.32 (0.26-0.39)	1700	2887818	5.89 (5.62-6.18)

Supplementary Table 9. Adjusted hazard ratios (HR) and 95% confidence intervals (CI) for outcomes in the pooled analyses (six seasons 2012/2013-2017/2018).

Covariates	Influenza (hospitalization/emergency room visit) Adjusted HR (95%CI)	Pneumonia (hospitalization/emergency room visit) Adjusted HR (95%CI)
Vaccine type (versus high-dose trivalent)		
Adjuvanted trivalent	1.05 (0.81-1.35)	1.02 (0.90-1.17)
Standard-dose trivalent	1.11 (1.03-1.19)	1.06 (1.04-1.09)
Standard-dose quadrivalent	1.15 (1.06-1.24)	1.04 (1.01-1.07)
Season (versus 2017/2018)		
2012/2013	0.28 (0.25-0.32)	0.93 (0.88-0.97)
2013/2014	0.16 (0.14-0.18)	0.84 (0.81-0.89)
2014/2015	0.66 (0.59-0.73)	0.94 (0.89-0.98)
2015/2016	0.17 (0.15-0.20)	0.83 (0.79-0.87)
2016/2017	0.43 (0.39-0.47)	0.88 (0.85-0.92)
Age (5-year increment)	1.22 (1.19-1.26)	1.31 (1.30-1.32)
Female sex (versus male)	0.92 (0.86-0.98)	0.77 (0.76-0.79)
Calendar time of vaccination	1.00 (1.00-1.00)	1.00 (1.00-1.00)
Hospitalization for pneumonia*	1.48 (1.30-1.68)	2.02 (1.96-2.08)
Diabetes*	1.00 (0.92-1.08)	1.04 (1.01-1.07)
Chronic obstructive pulmonary disease/asthma*	1.14 (1.05-1.23)	1.48 (1.44-1.52)
Myocardial infarction*	0.90 (0.76-1.05)	0.90 (0.87-0.94)
Congestive heart failure*	1.19 (1.09-1.30)	1.34 (1.30-1.37)
Peripheral vascular disease*	0.92 (0.84-1.01)	1.01 (0.98-1.04)
Renal disease*	1.01 (0.91-1.13)	1.09 (1.05-1.13)
Malignancy*	0.98 (0.90-1.08)	1.10 (1.06-1.13)
Frail physical health*	1.20 (1.11-1.30)	1.72 (1.68-1.76)
Past use of bronchodilators*	1.34 (1.21-1.48)	1.27 (1.23-1.31)
Past use of corticosteroids*	1.42 (1.32-1.53)	1.37 (1.34-1.41)
Past use of anticholinergics*	1.32 (1.19-1.46)	1.48 (1.45-1.52)
Charlson index*	1.06 (1.03-1.10)	1.08 (1.07-1.09)
Number of previous ER visits*	1.48 (1.40-1.56)	1.18 (1.15-1.20)
Number of previous physician visits*	1.01 (1.00-1.01)	1.01 (1.01-1.01)
Health plan type (versus Comprehensive)		
Health Maintenance Organization	0.90 (0.72-1.12)	0.95 (0.89-1.01)
Preferred Provider Organization	1.13 (1.04-1.24)	0.93 (0.90-0.97)
Other	1.14 (0.92-1.42)	0.96 (0.9-1.02)
Employment status (versus Retiree)		
Active Full Time	1.27 (1.12-1.43)	0.98 (0.92-1.05)
Other	1.34 (1.04-1.73)	1.08 (1.01-1.16)
Spouse/dependent	0.93 (0.83-1.05)	1.04 (1.00-1.09)
Region (versus South)		
Northeast	0.79 (0.70-0.89)	0.91 (0.88-0.95)

North Central	0.99 (0.89-1.1)	1.01 (0.98-1.04)
West	0.76 (0.65-0.88)	0.90 (0.86-0.94)
Urban area (versus rural)	1.20 (1.07-1.34)	0.94 (0.91-0.97)
Pneumococcal vaccine **	NA	1.00 (0.98-1.02)

*12 months before vaccination date. ** Any time before vaccination date. The models include all data available during the 2012/2013 and 2017/2018 seasons.

Supplementary Table 10. Adjusted relative vaccine effectiveness (RVE) and 95% confidence intervals (CI) for outcomes, using the high-dose trivalent vaccine as the reference group.

Season/Vaccine type	Influenza (hospitalization/emergency room visit)		Pneumonia (hospitalization/emergency room visit)	
	RVE	95% CI	RVE	95% CI
**All seasons**				
Adjuvanted trivalent	-1.8	-35.1 to 23.4	-3.9	-16.6 to 7.3
Standard-dose trivalent	-10.3	-18.2 to -2.8	-5.0	-7.2 to -2.9
Standard-dose quadrivalent	-15.6	-25.8 to -6.2	-5.8	-8.9 to -2.9
**2017-2018**				
Adjuvanted trivalent	10.4	-24.5 to 35.6	0.1	-16.1 to 14.0
Standard-dose trivalent	14.9	-5.8 to 31.5	-2.1	-12.3 to 7.2
Standard-dose quadrivalent	-3.4	-19.1 to 10.2	-3.9	-11.1 to 2.9
**2016-2017**				
Adjuvanted trivalent	-8.1	-92.5 to 39.3	-2.8	-25.0 to 15.5
Standard-dose trivalent	-5.8	-25.1 to 10.5	-4.7	-10.5 to 0.7
Standard-dose quadrivalent	-5.6	-26.8 to 12.0	-0.2	-6.4 to 5.6
**2015-2016**				
Standard-dose trivalent	-25.2	-57.4 to 0.5	-5.8	-11.0 to -0.9
Standard-dose quadrivalent	-21.7	-60.3 to 7.6	-4.2	-10.6 to 1.7
**2014-2015**				
Standard-dose trivalent	-6.0	-19.4 to 5.9	-2.9	-7.8 to 1.7
Standard-dose quadrivalent	-19.0	-39.5 to -1.5	-4.6	-11.6 to 1.9
**2013-2014**				
Standard-dose trivalent	-33.8	-74.5 to -2.5	-5.9	-11.3 to -0.8
Standard-dose quadrivalent	-95.0	-206.4 to -24.1	-22.1	-34.9 to -10.5
**2012-2013**				
Standard-dose trivalent	-38.1	-71.6 to -11.2	-6.6	-12.2 to -1.2

The models include all data available during the 2012/2013 and 2017/2018 seasons.
