# Supplementary material for: Relative effectiveness of influenza vaccines in elderly persons in the United States, 2012/2013-2017/2018 seasons
Source: NPJ Vaccines. 2021 Aug 24;6:108. doi: 10.1038/s41541-021-00373-w (PMC8385076; doi:10.1038/s41541-021-00373-w)
Supplement: Supplementary file 2 — Reporting Summary [file 41541_2021_373_MOESM2_ESM.pdf]

nature research

Corresponding author(s): DBPR NPJVACCINES-01145R

Last updated by author(s): Jul 5, 2021

Reporting Summary

Nature Research wishes to improve the reproducibility of the work that we publish. This form provides structure for consistency and transparency in reporting. For further information on Nature Research policies, see our Editorial Policies and the Editorial Policy Checklist.

Statistics

For all statistical analyses, confirm that the following items are present in the figure legend, table legend, main text, or Methods section.

n/a	Confirmed	
☐	☒	The exact sample size (n) for each experimental group/condition, given as a discrete number and unit of measurement
☐	☒	A statement on whether measurements were taken from distinct samples or whether the same sample was measured repeatedly
☐	☒	The statistical test(s) used AND whether they are one- or two-sided<br>*Only common tests should be described solely by name; describe more complex techniques in the Methods section.*
☐	☒	A description of all covariates tested
☐	☒	A description of any assumptions or corrections, such as tests of normality and adjustment for multiple comparisons
☐	☒	A full description of the statistical parameters including central tendency (e.g. means) or other basic estimates (e.g. regression coefficient) AND variation (e.g. standard deviation) or associated estimates of uncertainty (e.g. confidence intervals)
☐	☒	For null hypothesis testing, the test statistic (e.g. F, t, r) with confidence intervals, effect sizes, degrees of freedom and P value noted<br>*Give P values as exact values whenever suitable.*
☒	☐	For Bayesian analysis, information on the choice of priors and Markov chain Monte Carlo settings
☒	☐	For hierarchical and complex designs, identification of the appropriate level for tests and full reporting of outcomes
☒	☐	Estimates of effect sizes (e.g. Cohen's d, Pearson's r), indicating how they were calculated

Our web collection on statistics for biologists contains articles on many of the points above.

Software and code

Policy information about availability of computer code

Data collection	N/A
Data analysis	All analyses were performed using SAS version 9.4 (SAS Institute, Cary, NC).

For manuscripts utilizing custom algorithms or software that are central to the research but not yet described in published literature, software must be made available to editors and reviewers. We strongly encourage code deposition in a community repository (e.g. GitHub). See the Nature Research guidelines for submitting code & software for further information.

Data

Policy information about availability of data

All manuscripts must include a data availability statement. This statement should provide the following information, where applicable:

- Accession codes, unique identifiers, or web links for publicly available datasets
- A list of figures that have associated raw data
- A description of any restrictions on data availability

IBM MarketScan Medicare Supplemental Database is not in the public domain but is available to researchers at a cost. Data were used in compliance with privacy and confidentiality requirements.

Field-specific reporting

Please select the one below that is the best fit for your research. If you are not sure, read the appropriate sections before making your selection.

☒ Life sciences ☐ Behavioural & social sciences ☐ Ecological, evolutionary & environmental sciences

For a reference copy of the document with all sections, see nature.com/documents/nr-reporting-summary-flat.pdf

Life sciences study design

All studies must disclose on these points even when the disclosure is negative.

Sample size	In the pre-study feasibility assessment, we performed power calculations for two-by-two influenza vaccine comparisons. In the 2016/2017 season, we estimated a sample size of 330 thousand individuals, 4% of them exposed to the adjuvanted trivalent vaccine (smallest group) and the remaining distributed in the other three vaccine groups; and we assumed a hospitalization rate for influenza among seniors (≥65 years) in the US as 819/100,000. We concluded that the estimated sample size would allow us to detect a hazard ratio as small as 1.55 with more than 80% power for the comparison of the trivalent adjuvanted versus any other influenza vaccine.
Data exclusions	We required that individuals had continuous enrollment in their medical and pharmacy insurance plans for 12 months before the vaccination date to have baseline information on comorbidity and other factors. We excluded individuals residing in nursing homes or receiving hospice care on the vaccination date because their medical encounters may not be reliably captured. Those who had an influenza diagnosis before the time zero within each study year were also excluded.
Replication	Replication is possible upon the purchase of the IBM MarketScan Medicare Supplemental Database.
Randomization	N/A
Blinding	N/A

Reporting for specific materials, systems and methods

We require information from authors about some types of materials, experimental systems and methods used in many studies. Here, indicate whether each material, system or method listed is relevant to your study. If you are not sure if a list item applies to your research, read the appropriate section before selecting a response.

Materials & experimental systems

n/a	Involved in the study	
☒	☐	Antibodies
☒	☐	Eukaryotic cell lines
☒	☐	Palaeontology and archaeology
☒	☐	Animals and other organisms
☒	☐	Human research participants
☒	☐	Clinical data
☒	☐	Dual use research of concern

Methods

n/a	Involved in the study	
☒	☐	ChIP-seq
☒	☐	Flow cytometry
☒	☐	MRI-based neuroimaging
